# The Impact of Social Determinants of Health, Namely Financial Assistance, on Overall Survival in Advanced-Stage Non-Small Cell Lung Cancer Patients

**DOI:** 10.7759/cureus.36355

**Published:** 2023-03-19

**Authors:** Andreas Bello, Neeharika S Makani

**Affiliations:** 1 Research, Comprehensive Hematology Oncology, St. Petersburg, USA; 2 Research, Nova Southeastern University Dr. Kiran C. Patel College of Osteopathic Medicine, Davie, USA; 3 Oncology, Comprehensive Hematology Oncology, St. Petersburg, USA

**Keywords:** financial burden, financial assistance, social determinants of health (sdoh), overall survival (os), non-small cell lung cancer (nsclc)

## Abstract

Purpose: Non-small cell lung cancer (NSCLC) is the most prevalent form of lung cancer. Studies have evaluated the association of social determinants of health (SDH) with outcomes in early-stage NSCLC. These studies have shown statistically and clinically significant associations between overall survival (OS) and other SDH (e.g marital status, educational attainment).The aim of our study was to better understand the role of various SDH on OS in advanced-stage NSCLC patients in a community oncology practice in Florida.

Methods: In this retrospective study, 125 patients with stage III and IV NSCLC were identified between January 1, 2014, and December 31, 2018. We performed Pearson’s chi-square and Kruskal-Wallis test to evaluate the association between median OS and several independent variables, including; gender, race, marital status, insurance status, living status, receiving financial assistance (FA), alcohol use, and smoking histories. OS is defined as the date of diagnosis up to the date of death. Other confounders that were analyzed included histology, treatment modality, comorbidities, and performance status of the patients.

Results: Our results demonstrated that patients receiving FA had nearly a two-fold increase in median OS compared to patients without FA (median OS = 1.01 years vs. 0.545 years, respectively; p = 0.012).

Conclusion: Overall, this study highlighted the importance of reducing the financial burden of advanced-stage NSCLC on patients and how FA impacts patient outcomes. However, future prospective cohort studies with a larger sample size are warranted to identify other SDH, as well as the underlying mechanisms affecting median OS, in patients with advanced-stage NSCLC.

## Introduction

Non-small cell lung cancer (NSCLC) is the most common type of lung cancer, affecting nearly 80% of all lung cancer patients [1]. Adenocarcinoma and squamous cell carcinoma are the two most prevalent subtypes of NSCLC. Adenocarcinomas, the most common type of lung cancer in non-smokers, originate in mucus-secreting cells and affect smokers and non-smokers alike. In contrast, squamous cell carcinomas originate from squamous cells in the central lungs or airways and are more heavily associated with smoking history [1]. In terms of prognostic outcomes for advanced-stage NSCLC, the five-year survival rate is <10%. Also, 40% of all diagnosed NSCLC patients present with stage IV at the time of diagnosis [2]. Tethering the high prevalence of NSCLC among lung cancer patients with poor prognoses establishes a critically important research agenda for factors affecting overall survival (OS) among such patients.

Smoking is putatively associated with lung cancer development and continues to influence poor prognosis. Additionally, even after adjusting for smoking habits, low socioeconomic status (SES) groups had an increased risk of developing lung cancer [3]. Various social determinants of health (SDH) (e.g. SES) have been shown to affect patients’ OS rates. Research has shown that patients in lower-income areas with early-stage NSCLC had shorter six-year cancer-specific survival (CSS) compared to patients living in high-income areas [4]. Additionally, educational attainment has also been shown to impact patients’ clinical outcomes. For example, patients living in areas with a higher proportion of residents with a high school diploma had improved disease outcomes (i.e. OS) compared to patients in areas with a lower proportion of diploma-earning residents. Furthermore, increased survival rates for patients living in areas with a higher percentage of residents possessing bachelor’s degrees were observed [4]. Additionally, a comparative study found that the association between SES and cancer survival is weaker in Ontario, Canada, than it is in the United States (US) [5]. This study demonstrated that the healthcare system within a county, which directly impacts the type of insurance and care a patient is able to obtain, is also an important factor in clinical outcomes. Patients with low SES in Canada have higher survival rates compared to patients in the US with low SES. 

Other SDH, such as marital status and gender, have been linked to a greater risk of death or serve as protective factors against poor clinical outcomes for patients with NSCLC. For example, one study has shown that not only is marriage associated with longer survival and increased quality of life (QOL) [6], but unmarried patients are at a significantly higher risk of being diagnosed with late-stage metastatic lung cancer. Additionally, unmarried lung cancer patients are also undertreated [7]. Research has shown that a widowed status of the patient portends a more negative patient outcome. Widowed patients with surgery were found to be at greater risk of death across all stages compared to married and unmarried counterparts [8]. Additionally, widowed males had an increased risk of death compared to married males [9]. Together, these studies show that social support in the form of marital status impacts CSS. Studies have also demonstrated that a patient’s gender is another important variable to analyze when investigating NSCLC mortality rates. One study concluded that males with lung cancer had significantly higher mortality rates than females. Interestingly, the researchers also looked at the intersection of marital status and gender, concluding that single females had better OS than single males. Furthermore, it was found that married males had lower OS than married females [10]. This study highlighted the complex interplay between gender and marital status. 

Another factor outside of SDH that influences a patient’s OS is the treatment modality that a patient is able to receive. Chemotherapy and radiation therapy are the main treatment options available to patients with advanced-stage lung cancer. Chemotherapy is typically taken orally or by injection and exposes the whole body to cancer-fighting drugs. Immunotherapy is also a type of systemic therapy that has recently been approved for use in patients with advanced-stage lung cancer. Meanwhile, radiation therapy is a local treatment that uses high-energy waves to target the affected area alone [1]. Patients diagnosed with stage III NSCLC are usually candidates for combination therapy with systemic therapy and radiation therapy. This combination approach has been shown to improve patient survival. One study concluded that the use of chemotherapy and sequential radiation therapy increased the proportion of five-year survivors of stage III NSCLC by 2.8 compared to radiotherapy alone [11]. Another study found that the addition of chemotherapy to three-dimensional conformal radiation therapy (3D-CRT) showed a survival advantage compared to 3D-CRT alone [12]. In conjunction, these studies reveal that an aggressive, multimodal treatment approach appears to be best suited for patients with advanced NSCLC. Patients with stage IV disease are usually candidates for systemic therapy and radiation therapy is only reserved for palliative purposes.

## Materials and methods

Study population

**Figure 1:**
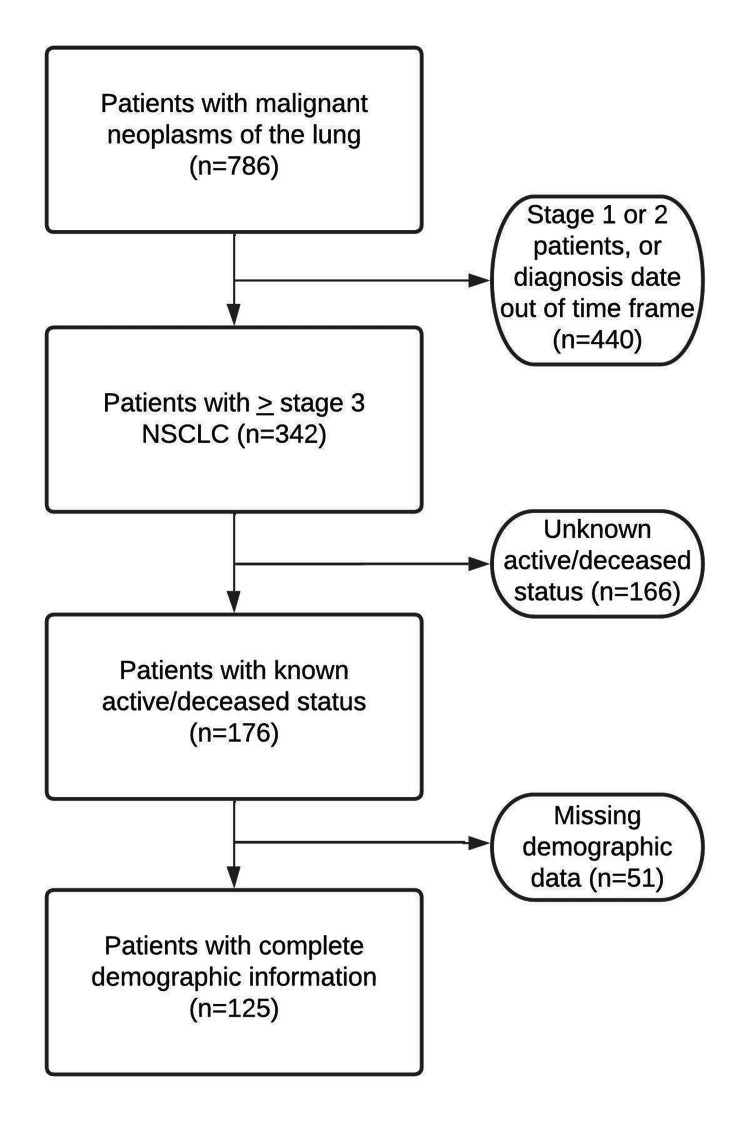
Study population inclusion process

The study population included 125 patients undergoing treatment for advanced-stage NSCLC at the Comprehensive Hematology Oncology clinic in St. Petersburg, Florida, US. Baseline demographic data, clinical characteristics, and outcome data were gathered from the OncoEMR system (The Flatiron Health, New York City, New York, US). Patients diagnosed with NSCLC between January 1, 2014, and December 31, 2018, were initially identified. Of the 786 patients found to have malignant neoplasms of the lung, 342 patients had advanced-stage NSCLC (stage III or stage IV). One hundred and sixty-six patients were excluded from the study for having an unknown survival status. Of the 176 patients with confirmed survival data, 51 patients had missing data and thus were excluded from the study. The baseline demographic and clinical characteristics of the study population are reported in Figure 1.

Measures

The social determinants that were analyzed in the study included the following: gender, race, marital status, insurance status, living status, receiving financial assistance (FA), alcohol use, and smoking history. Data were abstracted from initial clinical intake forms that all patients completed after their first oncological visit. Data collected from the intake forms included medical conditions such as cardiac disease, kidney problems, etc. A validated comorbidity scale, known as simplified comorbidity scores (SCS), is specifically used for patients with NSCLC and was utilized to help stratify patients by their comorbidity risk [13]. The scale is weighted in terms of the magnitude of the comorbidity’s effect on NSCLC disease outcome. The SCS includes seven comorbidities: tobacco consumption, diabetes mellitus, renal insufficiency, respiratory/cardiovascular/neoplastic comorbidity, and alcoholism. The weights for the comorbidities are: 7, 5, 4, 1, 1, 1, 1, respectively. The number of patients within each SCS range was also found (Table 1). The Eastern Cooperative Oncology Group (ECOG) performance status data were obtained from the first clinical note as documented by the physician. Most FA given to patients in this study population were through cancer foundations, grants, and pharmaceutical manufacturing companies.

**Table 1:** Simplified comorbidity scores (SCS) range

Range	0-7	8-9	10-11	12+
No. of patients	28	76	1	20

Statistical analysis

Univariate and bivariate analyses were performed on the total patient population (n=125). The independent variables that were studied are listed in Table 2. The main outcome variable was OS, defined as the date of diagnosis to the date of death. OS was analyzed in both a categorical and continuous fashion. For categorical analyses, Pearson’s chi-square test of significance was used to calculate statistical significance with a p-value of less than 0.05 being considered statistically significant. Statistical analyses were performed with the software “R” version 3.6.3 (2020, R Core Team, Vienna, Austria).

**Table 2:** Baseline demographic and clinical characteristics of the study population FA: financial assistance; HMO: health maintenance organization; PPO: preferred provider organization; ECOG: Eastern Cooperative Oncology Group; PS: performance scale

n= 125	No. (%)	p-value for Chi-square test	p-value for Kruskal Wallis test
Gender		0.304	0.223
Male	74 (59.2%)		
Female	51 (40.8%)		
Race		0.0145	0.0232
White	111 (88.8%)		
African-American	9 (7.2%)		
Other	5 (4%)		
Marital Status		0.150	0.174
Married	70 (56%)		
Divorced	15 (12.0%)		
Widowed	20 (16%)		
Single	20 (16%)		
Living Status		0.108	0.261
Alone	23 (18.45%)		
With	102 (81.6%)		
Receives FA		0.051	0.0122
Yes	64 (51.2%)		
No	61 (48.8%)		
Insurance Status		0.420	0.206
HMO	82 (65.6%)		
PPO	9 (7.2%)		
Medicare	32 (24.8%)		
Medicaid	1 (0.8%)		
Self Pay	1 (0.8%)		
Alcohol History		0.434	0.722
Denies use	67 (53.6%)		
Heavy	19 (15.2%)		
Social	28 (22.4%)		
Former	11 (8.8%)		
Smoking History		0.900	0.532
Never	13 (10.4%)		
Current	53 (42.4%)		
Former	59 (47.2%)		
Histology		0.731	0.646
Adenocarcinoma	70 (56%)		
Squamous	48 (38.4%)		
Other	7 (5.6%)		
Treatment Modality		0.840	0.331
Systemic Therapy Alone	61 (48.8%)		
Systemic Therapy Plus Radiation	58 (46.4%)		
Radiation Therapy Alone	4 (3.2%)		
Other	2 (1.6%)		
Simplified Comorbidity Score		0.147	0.0795
<8	72 (57.6%)		
>8	53 (42.4%)		
ECOG-PS (n=102)		0.901	0.345
0	29 (28.43%)		
1	62 (60.78%)		
2	11 (10.78%)		

**Figure 2:**
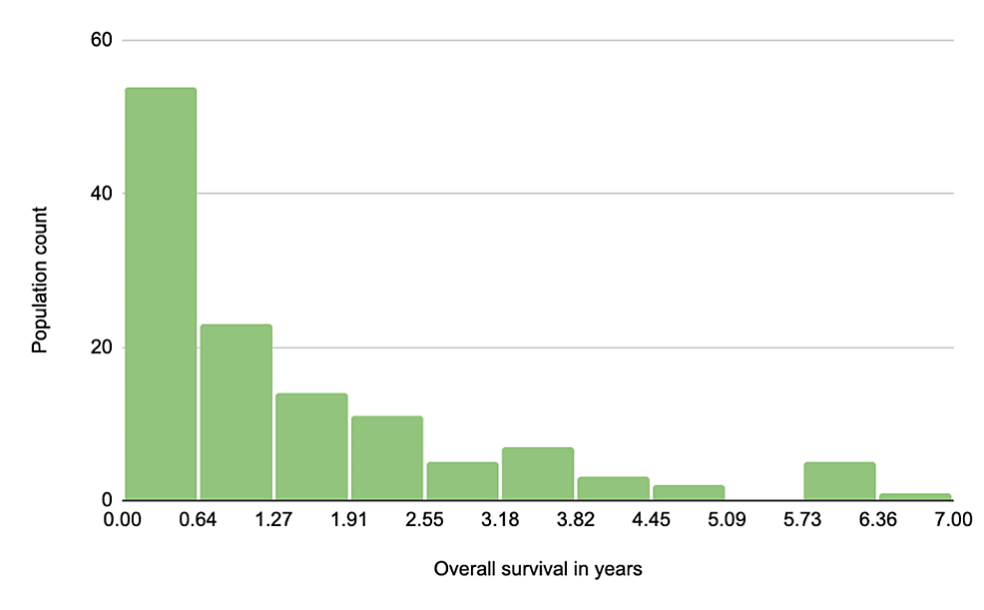
Population distribution of overall survival

Prior to conducting the continuous analyses, the Shapiro-Wilk test of normality was performed to determine if the study population had a normal distribution in regard to OS. The histogram resulting from the Shapiro-Wilk test of normality is shown in Figure 2. Due to the non-normal nature of the study population, the Kruskal-Wallis test was performed to determine the associations between each independent variable on OS. When running categorical tests, patients were categorized into two groups: (a) OS of less than one year or (b) OS greater than one year. For continuous tests, OS for each patient was determined by their date of diagnosis up to their date of death. For patients who were still active while the study was conducted, a set date of November 1, 2020, was used as the endpoint of their OS.

## Results

Of the total study population (n=125), 60% identified as male with a mean age of 73 years for the study population. The majority of patients (89%) identified as white; 56% were married, and 81% lived with someone. Sixty-six percent of patients had a health maintenance organization (HMO) insurance plan, and 51% of patients obtained FA to help with treatment care costs. Forty-seven percent of patients identified as former smokers and 54% denied any alcohol use. Regarding clinical characteristics, the majority of patients (56%) were diagnosed with adenocarcinoma. Forty-nine percent of patients were treated with systemic therapy alone (chemotherapy with or without immunotherapy), and 50% of patients received both systemic and radiation therapy for the treatment of their lung cancer. The median comorbidity score for the total patient population was 8. The majority of patients (61%) in this study presented with an ECOG performance status of 1. The median OS for the total patient population was 0.756 years.

In categorical analyses, an important trend for significance was found between patients who did and did not receive FA (p = 0.0509). Patients who received FA were more likely to live longer as opposed to patients that did not receive FA (odds ratio = 2.41, 95% confidence interval (CI) 1.18, 4.96). It was also found that there is a significant association between race and OS. 

**Figure 3:**
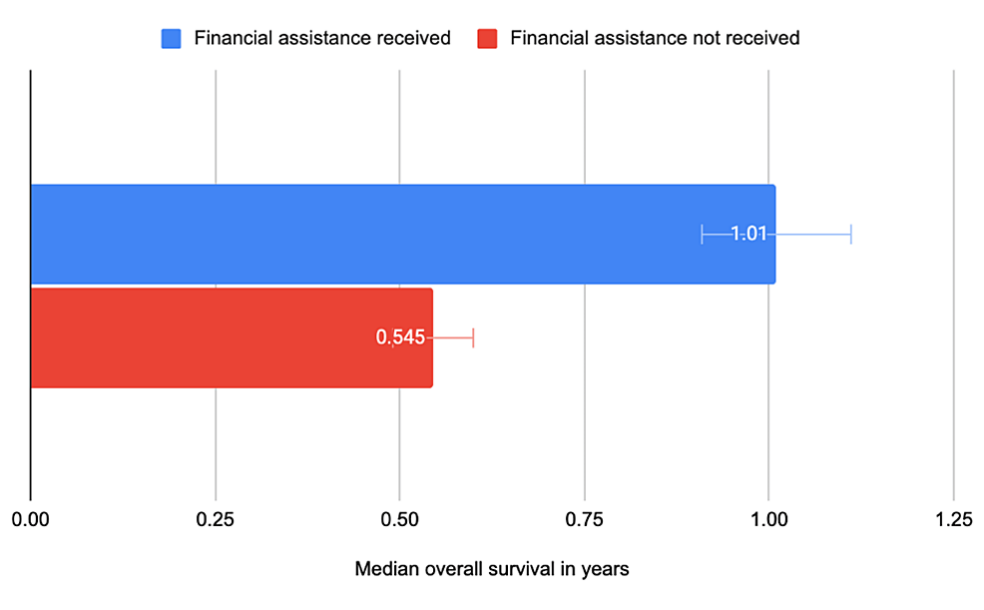
Financial assistance status vs median overall survival (OS)

**Figure 4:**
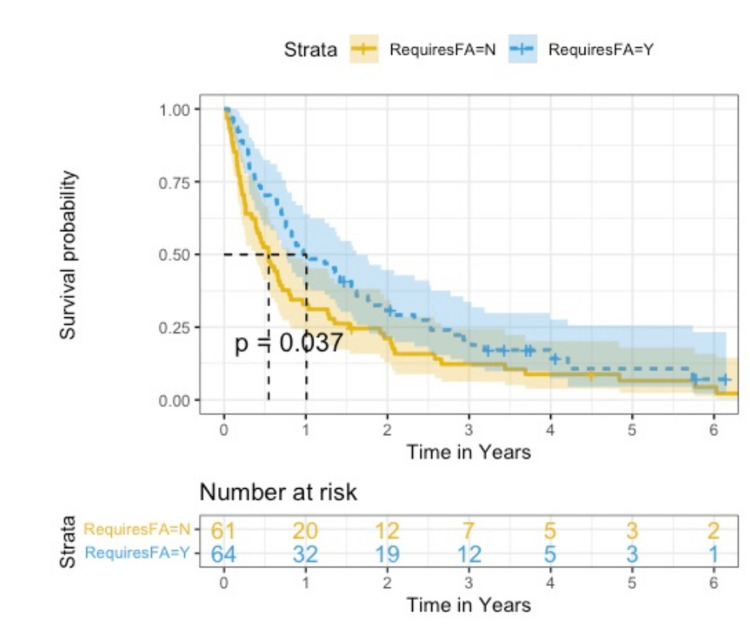
Kaplan-Meier curve plotting financial assistance against survival probability FA: financial assistance; Y: yes; N: no

When data were analyzed in a continuous fashion, there was again a statistically significant difference found in regard to FA (p = 0.013). For patients who did receive FA, the median OS was 1.01 years compared to a median OS of 0.545 years in patients who did not receive FA (Figure 3). A Kaplan-Meier survival curve was generated by plotting “receiving FA” against the probability of survival (Figure 4). This Kaplan-Meier curve serves as a visual representation of the estimated median survival probability of patients who received FA versus those who did not. The x-axis represents the survival time in years. The blue line represents the probability of survival of patients who received FA. Whereas, the yellow line represents the probability of survival of patients who did not receive FA. Importantly, this curve shows that patients who received FA had a 50% chance of survival of 1.01 years (95% CI 0.756, 1.620). Meanwhile, patients who did not receive FA had a 50% chance of survival of 0.545 years (95% CI 0.384, 0.816) (p = 0.037). No other statistically significant differences were found. However, there was a trend of statistical significance when analyzing the data based on the treatment modality the patient received. The median OS for patients who received systemic therapy alone had an OS of 0.567 years, whereas patients who received both systemic therapy and radiation therapy had a median OS of 0.855 years (p = 0.066).

## Discussion

This retrospective cohort study used univariate and bivariate analyses to investigate the associations between a multitude of social determinants on OS in advanced-stage NSCLC patients. Our study found that patients who received FA lived significantly longer compared to those who did not receive assistance. At first glance, these results may appear to contradict previously mentioned studies that showed that cancer patients with lower SES have worse clinical outcomes, including a shorter OS compared to patients with higher SES . However, in interpreting our results, it is important not to conceptually conflate FA with SES. Receiving FA eases financial burden, which has been shown to improve patients’ QOL. This is the first study demonstrating that relieving financial burden actually improves OS in patients with advanced NSCLC.

Cost of care is an important dimension to evaluate when discussing healthcare-related financial burden. One study investigated lung cancer costs in patients insured by Medicare and found that there has been a steady increase in the cost of cancer-related treatment for advanced lung cancer [14]. In addition, research has found that a two-year out-of-pocket healthcare cost for Medicare beneficiaries with cancer was $4,727 versus $3,209 for those without cancer [15]. The cost comparison shows a 47% increase in out-of-pocket costs for patients with cancer compared to those without. Such out-of-pocket costs reveal the financial burden that cancer patients face. Financial burden has been shown to have a negative psychological impact on patients. For example, compared to those with no financial hardships, patients who reported “a lot” of financial problems due to their cancer care were significantly more likely to rate their physical and mental health lower. Those patients also had significantly poorer satisfaction with their social activities and relationships [16]. Patient satisfaction for their oncologic care was also lower in patients who had a higher financial burden. Importantly, there is a significant difference in patient satisfaction scores between low financial burden and high financial burden (3.89 vs. 3.55; p < .01) [17].

While financial burden related to treatment has a negative impact on psychosocial health, there also exists a critical interplay between financial burden and factors that may influence OS. The interplay, for example, between financial burden and adherence to medications has been highlighted by numerous studies. Previous literature has demonstrated poor treatment compliance in patients with a higher financial burden. Furthermore, as a matter of public health concern, patients have shown decreased medication adherence as the average cost of cancer drugs increased nearly 10-fold for certain cancer therapies [18]. In a cross-sectional survey, 45% of participants committed a form of nonadherence due to cost. Additionally, 22% of patients took less than their prescription stated, and 27% did not refill the prescription at all [19]. Furthermore, compared to adherent participants, non-adherent participants were significantly more likely to cut spending on essentials like food and clothing [19]. Moreover, another study found that patients with higher financial burdens cut down on leisure spending by 68%, reduced spending on food and clothing by 48%, and 46% of those patients used savings to pay for out-of-pocket expenses [20].

Additionally, studies have shown an association between high financial burden and poor QOL among cancer patients [21]. A better QOL was associated with fewer perceptions of poor quality of care [22]. Patients’ own perception of their financial difficulties has also been shown to be strongly correlated with their satisfaction in QOL studies. In addition, one study investigated the association between QOL and FB in breast cancer survivors. The scale used was the QOL-Breast Cancer Survivors (QOL-BCS), and financial burden was measured using the Breast Cancer Finances Survey (BCFS). The study found that on the QOL-BCS, participants reported a significantly negative decline in motivation, productivity, and quality of work. Furthermore, an increase in economic events was found to be significantly associated with poorer QOL across all time points in the study [23]. Furthermore, cancer survivors with financial burdens were found to have significantly lower mental composite scores, with an increased chance of depressed mood [24]. Our study demonstrates that lessening financial burden by way of receiving FA improved OS in this advanced-stage NSCLC patient population. These patients may have had better treatment compliance, better perception of their quality of care, and/or improved QOL.

As our study is retrospective, a key limitation includes missing data. As can be seen from Figure 1, 51 (28.98%) patients were ineligible for the study due to insufficient demographic information alone. Additionally, all of the social determinant measures and comorbidities were obtained from patient questionnaires, and thus, the results may be skewed due to possible recall bias. Furthermore, the study population consisted of mostly White, Floridian patients with advanced-stage NSCLC from one community center, which limits the generalizability of these results to other races/ethnicities, geographic locations, and types of cancer. The proportion of patients identifying as White (89%) also reveals that the significant result obtained between race and OS is skewed.

Our study emphasizes the need for FA, especially in patients with advanced-stage malignancies. As the US healthcare system is privatized, future research comparing the OS of advanced-stage cancer patients between public and privatized healthcare between countries would be interesting. Prospective studies investigating the relationship between OS, QOL, and treatment adherence with financial burden should be conducted with a more diverse patient population, as well. Finally, future prospective studies comparing early-stage vs. advanced-stage NSCLC patients with respect to the impact of social determinants on OS should be considered.

## Conclusions

Our study concluded that receiving FA has a significant positive association with OS in advanced-stage NSCLC patients. Other social determinants in this study were not significant factors in OS. This may be due to the advanced nature of the cancer itself. Therefore, future studies investigating FA status and OS are warranted. Additionally, it is important to note the limitation of missing data from the other excluded patients due to incomplete demographic information within the electronic medical record (EMR) system. Importantly, this raises the need for comprehensive EMR management and documentation across community and academic centers alike. Ultimately, this study highlights the importance of reducing the financial burden for patients suffering from advanced-stage malignancies and the need for more FA to be available to cancer patients.
